# Peristaltic Creeping Flow of Power Law Physiological Fluids through a Nonuniform Channel with Slip Effect

**DOI:** 10.1155/2015/152802

**Published:** 2015-07-05

**Authors:** M. K. Chaube, D. Tripathi, O. Anwar Bég, Shashi Sharma, V. S. Pandey

**Affiliations:** ^1^Department of Applied Mathematics, Echelon Institute of Technology, Faridabad 121002, India; ^2^Department of Mechanical Engineering, Manipal University Jaipur, Rajasthan 303007, India; ^3^GORT Engovation-Aerospace and Biomechanics, Southmere Avenue, Bradford BD7 3NU, UK; ^4^Department of Mathematics, Indian Institute of Technology, Roorkee 247667, India; ^5^Department of Physics, National Institute of Technology Delhi, Delhi 110040, India

## Abstract

A mathematical study on creeping flow of non-Newtonian fluids (power law model) through a nonuniform peristaltic channel, in which amplitude is varying across axial displacement, is presented, with slip effects included. The governing equations are simplified by employing the long wavelength and low Reynolds number approximations. The expressions for axial velocity, stream function, pressure gradient, and pressure difference are obtained. Computational and numerical results for velocity profile, pressure gradient, and trapping under the effects of slip parameter, fluid behavior index, angle between the walls, and wave number are discussed with the help of *Mathematica* graphs. The present model is applicable to study the behavior of intestinal flow (chyme movement from small intestine to large intestine). It is also relevant to simulations of biomimetic pumps conveying hazardous materials, polymers, and so forth.

## 1. Introduction

The transportation of physiological fluids due to continuous wavelike muscle contraction and relaxation of physiological vessels such as the oesophagus, stomach, intestines, ureter and blood vessels (arteries, veins, capillaries, etc.), and other hollow tubes is known as* peristalsis* [1]. Peristalsis is used in many diverse applications in the human body. These include urodynamic conveyance from the kidneys to the bladder [2], swallowing of food through the esophagus, the movement of chyme in gastrointestinal tract, intrauterine fluid motion, and the flow of spermatozoa in the ductus efferentes of the male reproductive tract. Further applications include the movement of ovum in the female fallopian tube, transport of lymph in the lymphatic vessels, and the vasomotion of small blood vessels such arterioles, venules, and capillaries. These are all* internal* peristaltic mechanisms. In* biolocomotion*, earthworms also use peristalsis as an external motion achieving very efficient “geonautical” mobility, aided by the secretion of lubricating mucus. This also serves to subject the soil to continuous biological “pistons” forcing air through burrowed tunnels, promoting aeration and soil mixing, and encouraging mineralization of nutrients and their uptake by vegetation [3]. Roller and finger pumps also operate on this principle and furthermore modern micro- and nanorobots are exploiting peristaltic mechanisms [4].

The behavior of most of the physiological fluids is known to be non-Newtonian. A simple yet versatile rheological model is the* Ostwald-DeWaele power law model* which successfully simulates viscosity, shear thickening, and shear thinning effects. Representative studies deploying this model in peristaltic fluid dynamics include [5–9] wherein the effect of fluid behavior index on peristaltic pumping has been examined. Other researchers [10–18] have deployed alternative rheological models for peristaltic transport of non-Newtonian fluids including Eyring-Powell fluids [10], couple stress fluids [11], Williamson viscoelastic fluids [12], Eringen micromorphic models [13], fractional viscoelastic models [14], Oldroyd-B viscoelastic models [15], second-grade differential Reiner-Rivlin viscoelastic fluids [16], micropolar models [17], and Herschel-Bulkley yield-stress fluids [18]. These studies explored a variety of flow geometries and generally utilized the no-slip boundary condition at the walls. However, in real physiological systems, slip effects can arise at the walls, invalidating the classical Navier no-slip boundary condition. This modified boundary condition has been shown to exert a significant effect on transport phenomena in the near-wall region of biopolymeric sheet [19], gastric duct [20], and abnormal swallowing dynamics [21].

Kwang et al. [22] studied the peristaltic transport of a Newtonian fluid through a 2D microchannel where the slip effect is present. Ali et al. [23] investigated slip effects on the peristaltic transport of variable viscosity magnetic fluid. Hayat et al. [24] studied slip effect on the peristaltic motion of a third-order rheological fluid in an asymmetric channel. Ebaid [25] analyzed effects of magnetic field and wall slip conditions on the peristaltic transport of a Newtonian fluid in an asymmetric channel. Recently Tripathi et al. [26] studied slip effects in fractional viscoelastic Oldroyd gastric flows using a homotopy method, showing that pressure is decreased with increasing slip.

It has been pointed out by Charm and Kurland [27, 28] that the flow behavior of blood in vessels of small diameter (0.02 cm) and at low shear rates (<20 s^−1^) can be represented by a power law fluid. Also, it is found that physiological organs are generally nonuniform ducts [29, 30]. Remaining cognizant of these facts, in this paper we investigate* peristaltic transport of power law fluid in a nonuniform channel under a slip boundary condition*. The effects of slip parameter, fluid behavior index, angle between the walls, and wave number on pumping characteristics and trapping phenomenon are investigated numerically and depicted graphically.

## 2. Mathematical Formulation

**Figure 1:**
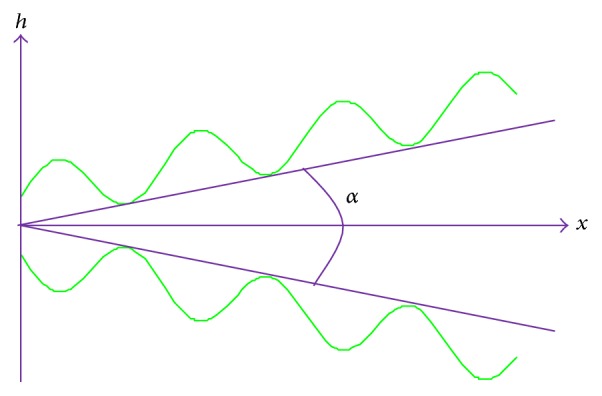
Geometry of nonuniform peristaltic channel.

We consider the peristaltic flow of power law fluid in a nonuniform channel under a hydrodynamic slip boundary condition (see Figure 1). Let the motion of the walls of the channel be governed by a sinusoidal nonuniform wave which is mathematically modelled as

(1) $$\begin{array}{c} h=a+x\tan\alpha +b\sin\left(\;\frac{2\pi x}{\lambda }\;\right), \end{array}$$

where *h*, *a*, *b*, *λ*, *x*, *α* are transverse vibration of the wall, half width of the channel, amplitude, wavelength, axial displacement, and angle between walls of channels, respectively. The sinusoidal nature of peristaltic waves is established in numerous clinical studies and we refer readers to the standard monograph Keener and Sneyd [31].

The governing equations of the motion of power law fluids (see, e.g., [6] for two-dimensional channel flow) are given by

(2) ∂u∂x+∂v∂y=0,ρu∂u∂x+v∂u∂y=−∂p∂x+∂τxx∂x+∂τyx∂y,ρu∂v∂x+v∂v∂y=−∂p∂y+∂τxy∂x+∂τyy∂y,

where *τ*_*xx*_, *τ*_*xy*_, *τ*_*yy*_ are the shear stress components and *ρ*, *u*, *v*, *y*, *p* are the fluid density, axial velocity, transverse velocity, transverse coordinate, and pressure, respectively.

We introduce the following dimensionless parameters:

(3) x′=xλ,y′=ya,u=u′c,v=v′cδ,h′=ha,ϕ=ba,p′=pan+1μcλ,δ=aλ,

where *c*, *δ*, *ϕ*, *μ* are the wave velocity, wave number, amplitude ratio, and viscosity, respectively, and *n* is the fluid behavior index (i.e., *n* < 1 is pseudoplastic and *n* > 1 is the dilatant fluid and *n* = 1 is the Newtonian fluid). Using the above nondimensional variables and taking into account long wavelength and low Reynolds number approximation, after dropping the primes, the governing equations for flow of a power law fluid reduce to

(4) ∂u∂x+∂v∂y=0,

(5) ∂p∂x=sign⁡∂u∂y∂∂y∂u∂yn,

(6) ∂p∂y=0,

where sign is a Signum function and is defined as

(7) $$\begin{array}{c} \mathrm{sign}\left(\;x\;\right)=\left\{\begin{array}{cc} -1, & \text{if }x<0\\ 0, & \text{if }x=0\\ 1 & \text{if }x>0. \end{array}\right. \end{array}$$

The nondimensional wall equation in the wave frame is

(8) $$\begin{array}{c} h=1+\frac{x}{\delta }\tan\alpha +\phi \sin2\pi x. \end{array}$$

Boundary conditions in the wave frame of reference are specified thus:

(9) ∂u∂y=0,at  y=0,

(10) u=−1∓β∂u∂yat  y=±h,

where *β*(=*L*/*a*) is the dimensionless slip parameter and *L* is the dimensional slip parameter.

## 3. Analytical Solutions

Integrating (5) with respect to *y* and using condition (9) we get

(11) $$\begin{array}{c} \frac{\partial u}{\partial y}=y^{1/n}\left(\;\mathrm{sign}\frac{\partial p}{\partial x}\;\right)\left(\;{\left|\;\frac{\partial p}{\partial x}\;\right|}^{1/n}\;\right). \end{array}$$

Again integrating (11) with respect to *y* and using condition (10) we get

(12) usign⁡∂p∂xnn+1∂p∂x1/n·y1+n/n−h1+n/n−1+nnβh1/n−1.

The stream function is defined, based on Cauchy-Riemann equations, as

(13) u=∂ψ∂y,v=−∂ψ∂x.

Using (12) and (13) we get

(14) ψsign⁡∂p∂xnn+1∂p∂x1/n·n2n+1y2n+1/n−yhn+1/n−n+1nβyh1/n−y.

The nondimensional volumetric flow rate in the wave frame is defined as

(15) $$\begin{array}{c} q=\overset{h}{\underset{0}{\int }}u\;dy={\left|\;\frac{\partial p}{\partial x}\;\right|}^{1/n}h^{\left(1+n\right)/n}\left\{\;\left(\;\frac{n}{2n+1}\;\right)h+\beta \;\right\}-h. \end{array}$$

The pressure gradient is obtained from (15) as follows:

(16) $$\begin{array}{c} \frac{dp}{dx}=-\frac{{\left(q+h\right)}^{n}}{h^{\left(1+n\right)}{\left\{\left(nh/\left(2n+1\right)\right)+\beta \right\}}^{n}}. \end{array}$$

Integrating (16) with respect to *x*, the* pressure difference across the axial line* is

(17) $$\begin{array}{c} p\left(\;x\;\right)-p\left(\;0\;\right)=-\overset{x}{\underset{0}{\int }}\frac{{\left(q+h\right)}^{n}}{h^{\left(1+n\right)}{\left\{\left(nh/\left(2n+1\right)\right)+\beta \right\}}^{n}}dx. \end{array}$$

## 4. Numerical Results and Interpretation

**Figure 2:**
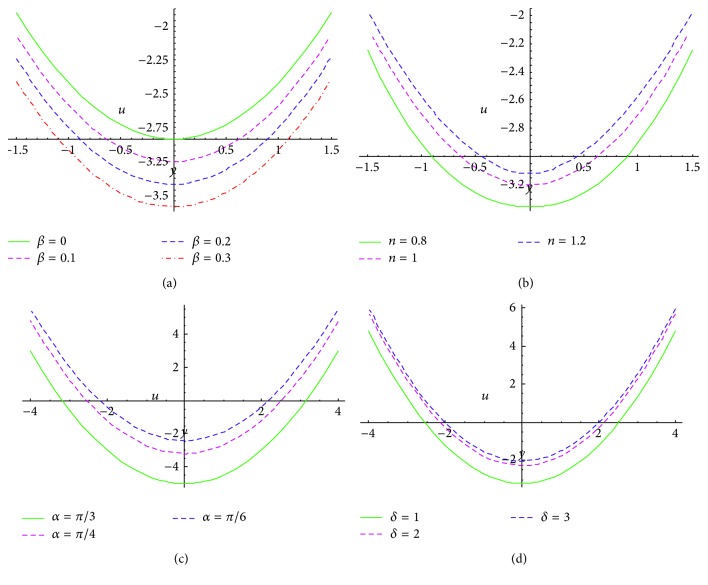
Velocity profiles (axial velocity versus transverse displacement) at *ϕ* = 0.5, ∂*p*/∂*x* = 1, *x* = 1 for (a) *n* = 1, *α* = *π*/4, *δ* = 1, and various values of slip parameter *β* = 0, 0.1, 0.2, 0.3, (b) *β* = 0.1, *α* = *π*/4, *δ* = 1, and various values of fluid behavior index *n* = 0.8,1, 1.2, (c) *β* = 0.1, *n* = 1, *δ* = 1, and various values of inclination of channel *α* = *π*/3, *π*/4, *π*/6, and (d) *β* = 0.1, *n* = 1, *α* = *π*/4, and various values of wave number *δ* = 1, 2, 3.

**Figure 3:**
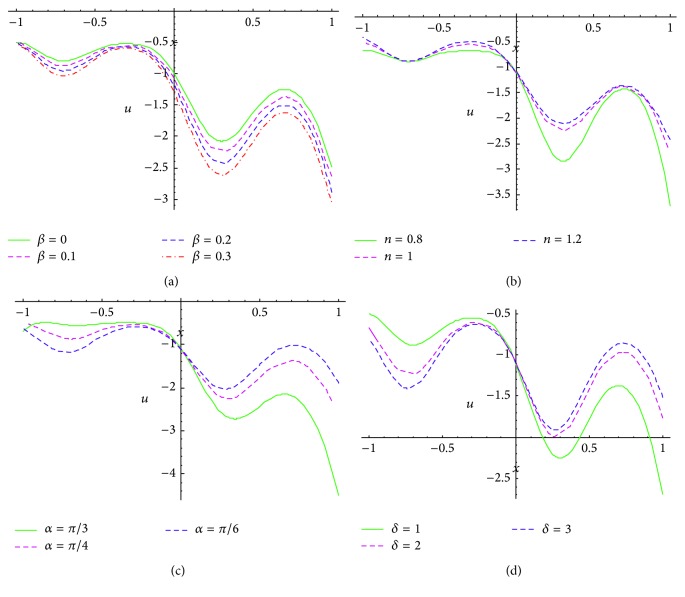
Axial velocity versus axial displacement at *ϕ* = 0.5, ∂*p*/∂*x* = 1, *y* = 1 for (a) *n* = 1, *α* = *π*/4, *δ* = 1, and various values of slip parameter *β* = 0, 0.1, 0.2, 0.3, (b) *β* = 0.1, *α* = *π*/4, *δ* = 1, and various values of fluid behavior index *n* = 0.8, 1, 1.2, (c) *β* = 0.1, *n* = 1, *δ* = 1, and various values of inclination of channel *α* = *π*/3, *π*/4, *π*/6, and (d) *β* = 0.1, *n* = 1, *α* = *π*/4, and various values of wave number *δ* = 1, 2, 3.

**Figure 4:**
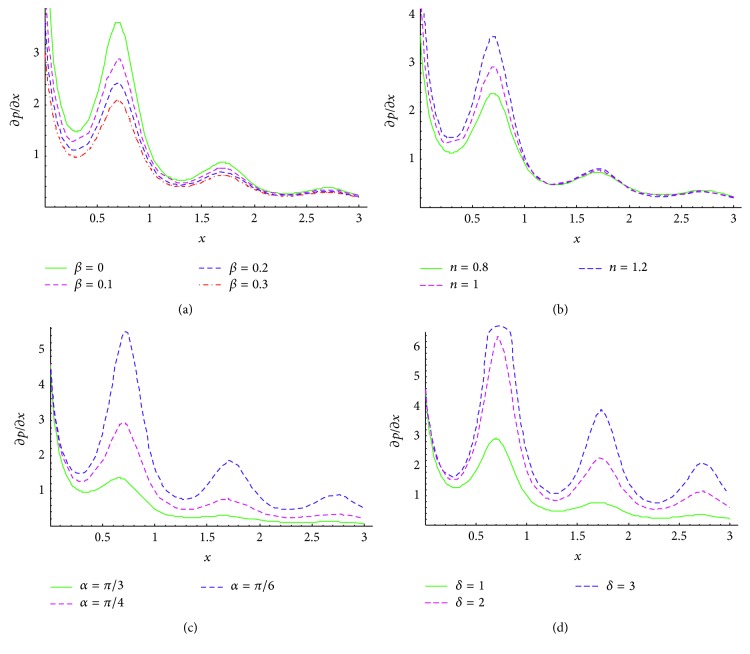
Pressure gradient versus axial displacement at *ϕ* = 0.5, *q* = 1 for (a) *n* = 1, *α* = *π*/4, *δ* = 1, and various values of slip parameter *β* = 0, 0.1, 0.2, 0.3, (b) *β* = 0.1, *α* = *π*/4, *δ* = 1, and various values of fluid behavior index *n* = 0.8, 1,1.2, (c) *β* = 0.1, *n* = 1, *δ* = 1, and various values of inclination of channel *α* = *π*/3, *π*/4, *π*/6, and (d) *β* = 0.1, *n* = 1, *α* = *π*/4, and various values of wave number *δ* = 1, 2, 3.

**Figure 5:**
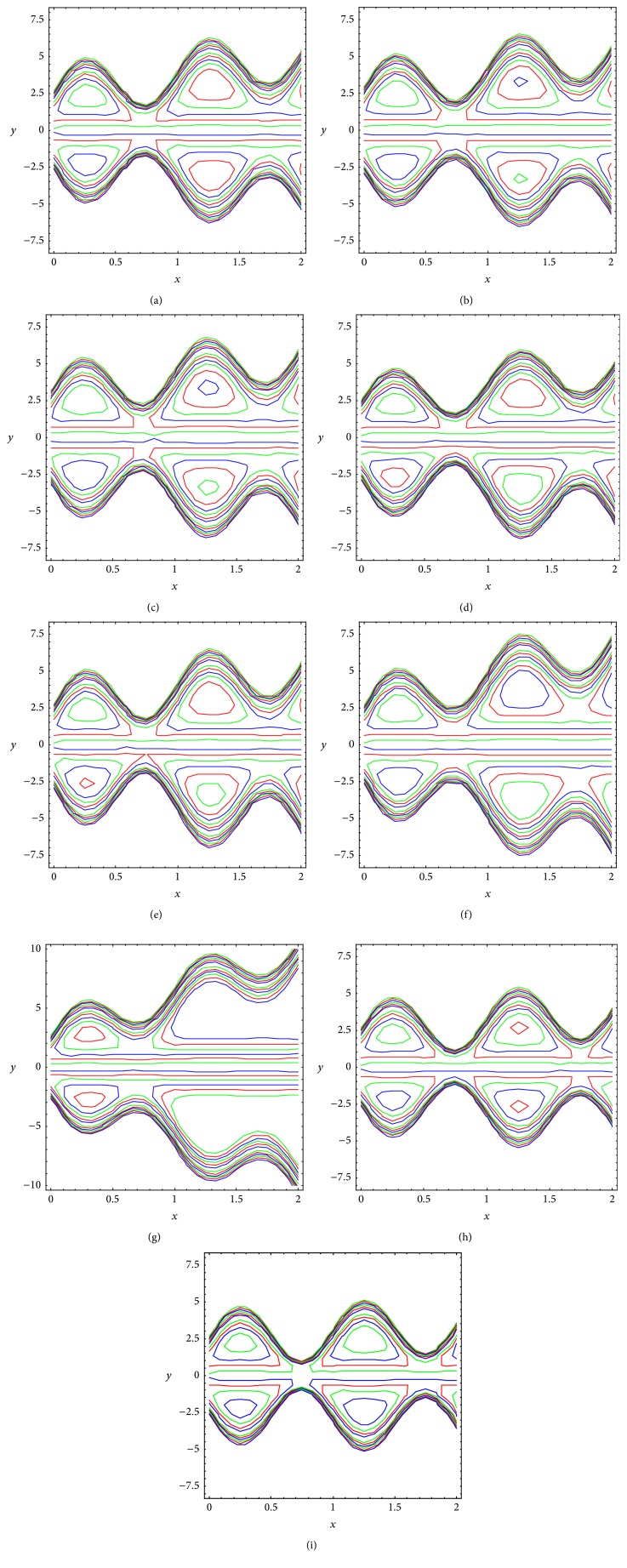
Streamlines in wave frame at *ϕ* = 0.3, *q* = 0.6 for (a) *β* = 0, *n* = 1, *α* = *π*/6, *δ* = 1, (b) *β* = 0.1, *n* = 1, *α* = *π*/6, *δ* = 1, (c) *β* = 0.2, *n* = 1, *α* = *π*/6, *δ* = 1, (d) *β* = 0, *n* = 0.8, *α* = *π*/6, *δ* = 1, (e) *β* = 0, *n* = 1.2, *α* = *π*/6, *δ* = 1, (f) *β* = 0, *n* = 1, *α* = *π*/4, *δ* = 1, (g) *β* = 0, *n* = 1, *α* = *π*/3, *δ* = 1, (h) *β* = 0, *n* = 1, *α* = *π*/6, *δ* = 2, and (i) *β* = 0, *n* = 1, *α* = *π*/6, *δ* = 3.

In this section, numerical calculations executed on* Mathematica* software are presented via graphs, that is, Figures 2–5.

We systematically study the effects of slip parameter (*β*), rheological fluid power index (*n*), angle between the walls (*α*), and wave number (*δ*) on the velocity profile, pressure gradient, and the trapping phenomenon.

Figures 2(a)–2(d) illustrate the velocity profiles (axial velocity versus transverse displacement). All plots exhibit a distinctly parabolic shape and are generally symmetric along the* transverse (y-)*axis.  Figure 2(a) depicts the effect of slip parameter on velocity profile at prescribed values of other physical parameters, *ϕ* = 0.5, ∂*p*/∂*x* = 1, *x* = 1, *n* = 1, *α* = *π*/4, *δ* = 1. Evidently the curves for velocity profile are* displaced downwards* when the magnitude of *β* increases from 0 to 0.3. The curve for *β* = 0 represents the velocity profile for a uniform no-slip channel. The slip boundary condition defined in (10) is a Navier modification of the conventional no-slip condition. In certain physiological fluids, a partial nonadherence of the fluid to a solid boundary is observed. This constitutes momentum or velocity slip. This has been observed over four decades ago in celebrated clinical physiological testing studies with Weissenberg rheogoniometry for both blood and intestinal liquids [32, 33]. As such, to provide a more realistic appraisal of actual peristaltic transport, a slip condition is advisable.  Figure 2(a) shows that as the slip parameter increases, the magnitude of the axial velocity (*u*) is evidently boosted. The fluid moves faster at the boundary with greater slip. This adds momentum to the near wall flow which is transferred to the core region also and generates a consistent acceleration in the flow. In the absence of the momentum slip effect (*β* = 0) the magnitude is suppressed. The implication is that, with a slip effect, the axial flow distribution receives a* nontrivial* modification (acceleration) which is generally ignored in the majority of peristaltic flow models, and this can influence the efficiency of the peristaltic pumping. It may further be noted that with* heat and species diffusion present* (not studied in the current analysis) thermal jump (slip) and solutal slip (mass slip) at the deformable boundaries can also be incorporated and this is being considered by the authors for future investigations.

Figure 2(b) illustrates the impact of fluid behavior index on velocity profile at fixed values *β* = 0.1, *α* = *π*/4, *δ* = 1, *ϕ* = 0.5, ∂*p*/∂*x* = 1, *x* = 1. It is found that the curve is displaced in an upward direction with increasing the value of *n*. The curve for *n* < 1 (*n* = 0.8) represents the velocity profile for pseudoplastic and for *n* > 1 (*n* = 1.2) represents the dilatant fluid and for *n* = 1 represents Newtonian fluid.  Figure 2(c) describes the velocity profile for various values of angle between the peristaltic walls (*α* = *π*/3, *π*/4, *π*/6) at fixed values *β* = 0.1, *n* = 1, *δ* = 1, *ϕ* = 0.5, ∂*p*/∂*x* = 1, *x* = 1. The curves of velocity profile move downwards with large inclination between the peristaltic walls.  Figure 2(d) shows the curve between axial velocity and transverse displacement for various values of wave number (*δ* = 1, 2, 3) at fixed value of *ϕ* = 0.5, ∂*p*/∂*x* = 1, *x* = 1, *β* = 0.1, *n* = 1, *α* = *π*/4. The magnitude of axial velocity increases with increasing the wave number.

Figures 3(a)–3(d) illustrate the evolution of axial velocity with axial displacement (longitudinal coordinate) for variation of the slip parameter (*β*), rheological fluid behavior index (*n*), channel inclination angle (*α*), and wave number (*δ*). Inspection of these figures confirms the sinusoidal nature of the axial flow in the direction of propagation of the peristaltic waves. In all these graphs the channel is diverging (*α* > 0). The axial velocity is generally enhanced in magnitude with greater wall slip effect (Figure 3(a)), and the amplitudes are progressively increased with progressive distance from the apex of the channel. With greater geometric divergence of the channel, the peristaltic wave is allowed to grow considerably and axial flow is substantially accelerated with increasing slip. Conversely with greater power law index, owing to an elevation in biofluid viscosity, the momentum in the propulsion is opposed and the axial velocity is depleted, as observed in Figure 3(b). Dilatant (*n* > 0) biofluids clearly propel slower than pseudoplastic (*n* < 0) biofluids.  Figure 3(c) reveals that as the channel apex angle (inclination) is increased, the axial velocity along the pumping direction is markedly accelerated again. Naturally with an expanding frontier to propel into, the waves grow and the biofluid accelerates. Finally in Figure 3(d), we find that, with greater wave number, the axial velocity magnitudes are enhanced for *x* > 0 whereas they are decreased for *x* < 0.

Figures 4(a)–4(d) show the pressure gradient across the axial displacement for different physical parameters. The pattern of pressure gradient is* nonlinear* and is opposite to the geometry of nonuniform peristaltic channel across the longitudinal axial line. It is apparent that pressure gradient is* maximized* at the point of contraction and* minimized* at the point of relaxation. Pressure enhances with distance between the walls. The effect of slip parameter on pressure gradient at fixed values of other physical parameters *ϕ* = 0.5, *q* = 1, *n* = 1, *α* = *π*/4, *δ* = 1 is shown in Figure 4(a). It is observed that pressure gradient* increases* with* increasing* the magnitude of *β*. The impact of fluid behavior index (*n*) on pressure gradient at fixed values *ϕ* = 0.5, *q* = 1, *β* = 0.1, *α* = *π*/4, *δ* = 1 is illustrated in Figure 4(b). It is found that the pressure gradient increases with fluid behavior index. Pressure gradient for pseudoplastic biofluid is a minimum and it is maximum for dilatant fluid.  Figure 4(c) shows the effect of inclination between the peristaltic walls on pressure gradient for various values of (*α* = *π*/3, *π*/4, *π*/6) at fixed values *ϕ* = 0.5, *q* = 1, *β* = 0.1, *n* = 1, *δ* = 1. It is found that the pressure gradient is maximum with small inclination and minimum with large inclination.  Figure 4(d) depicts the effect of wave number on pressure gradient at fixed value of *ϕ* = 0.5, *q* = 1, *β* = 0.1, *n* = 1, *α* = *π*/4. The pressure gradient once again is found to be enhanced with increasing the wave number.

Trapping is an interesting phenomenon in peristaltic motion in which an internally circulating bolus of fluid is formed by closed streamlines and this trapped bolus is pushed ahead along with the peristaltic wave. The effects slip parameter, power law index, angle between the walls, and wave number are illustrated with the help of contour plots (Figures 5(a)–5(i)) of streamlines. A general observation regarding the effects of slip parameter (*β*), fluid behavior index (*n*), angle between the walls (*α*), and wave number (*δ*) is that the trapped bolus increases in size as *β*, *n*, and *α* increase. However, the size of the trapped bolus decreases in size as *δ* increases. Evidently slip exerts a nontrivial influence on pressure gradient, velocity, and bolus magnitude and growth.

## 5. Conclusions

In this study the two-dimensional peristaltic flow of a power law physiological fluid with the effect of slip condition through a nonuniform channel has been investigated. On the basis of computational and numerical results, the main findings of the present study are as follows:

(i) Axial velocity across the transverse displacement is parabolic in nature and shifted in a downward direction increasing *β* and *α* and the converse behavior with *n* and *δ*.
(ii) Pressure gradient across the axial length increases with slip parameter, fluid behavior index, and wave number and decreases with increasing inclination between walls.
(iii) The size of trapped bolus increases with *β*, *n*, and *α* increase and decreases with *δ*.

The present study has ignored* curvature* effects of the physiological vessel which are important in clinical applications and also biomimetic pumps employed in chemical engineering. These introduce a Coriolis effect and can lead to secondary vortex effects. They have been studied by other authors [34] and will be addressed imminently.

## Nomenclature

*h*: Transverse vibration of the wall
*a*: Half width of the channel
*b*: Amplitude
*x*: Axial displacement
*u*: Axial velocity
*v*: Transverse velocity
*y*: Transverse coordinate
*p*: Pressure
*c*: Wave velocity
*n*: Fluid behavior index
*L*: Dimensional slip parameter
*q*: Volumetric flow rate in the wave frame
*δ*: Wave number
*ϕ*: Amplitude ratio
*μ*: Viscosity
*β*: Dimensionless slip parameter
*ψ*: Stream function
*λ*: Wavelength
*α*: Angle between walls of channels
*τ*_*xx*_, *τ*_*xy*_, *τ*_*yy*_: Shear stress components
*ρ*: Fluid density.
